# Comparative Metabarcoding of ITS1, ITS2, and Full‐Length ITS Reveals Marker‐ and Tissue‐Specific Variation in Fungal Community Profiling in Potato

**DOI:** 10.1002/pei3.70168

**Published:** 2026-06-02

**Authors:** Silvia Turco, Irene Giubilei, Lovely Mahawar, Angelo Mazzaglia, Benedicte Riber Albrectsen

**Affiliations:** ^1^ Dipartimento di Scienze Agrarie e Forestali Università degli Studi della Tuscia Viterbo Italy; ^2^ Department of Plant Physiology Umeå Plant Science Centre, Umeå University Umeå Sweden

**Keywords:** amplicon sequencing, ITS markers, metabarcoding, mycobiome, *Solanum tuberosum* L.

## Abstract

Accurate profiling of plant‐associated fungal and oomycete communities depends critically on the choice of ITS marker. Using Illumina sequencing for ITS1/ITS2 and PacBio HiFi sequencing for full‐length ITS (ITSf), we compared read recovery, taxonomic assignment depth, and diversity patterns across potato leaf and root tissues. ITS2 generally recovered more diverse and even community profiles in leaves, whereas ITSf recovered comparatively more even and taxonomically broad profiles in roots. In contrast, ITS1 showed high read recovery but produced strongly skewed compositional profiles and was frequently dominated by host‐derived sequences. Beta‐diversity analyses indicated that ITS marker choice was associated with substantial variation in observed community composition, while functional annotation highlighted communities composed of taxa associated with multiple ecological guilds, including endophytes and opportunistic pathogens such as *Cladosporium* and *Ilyonectria*. Overall, the results demonstrate that ITS marker choice strongly influences the observed structure and diversity of plant‐associated communities. ITS2 was generally more suitable for phyllosphere‐associated communities, whereas ITSf provided broader recovery of root‐associated taxa. Combining complementary markers therefore offers a more comprehensive representation of potato‐associated microbial eukaryotic communities than single‐marker approaches alone.

## 1 Introduction

Fungi are one of the largest groups of eukaryotic organisms, inhabiting nearly all ecosystems where they play essential ecological roles as decomposers, mutualists, and pathogens. They contribute to nutrient cycling, organic matter decomposition, and ecosystem functioning, while also influencing plant and animal health through both beneficial (endophytic and mycorrhizal) and pathogenic associations (Blackwell 2011; Gautam et al. 2022; Runnel et al. 2025). However, despite their vital ecological roles and wide‐ranging industrial applications, only a small fraction of fungal diversity has been formally described. This gap reflects both the vast unexplored diversity and the underappreciated potential of fungi as resources for biotechnology and ecosystem services (Hyde et al. 2019). Current estimates indicate that fewer than 5%–10% of all fungal species are known to science (Hawksworth and Lücking 2017; Hyde et al. 2019), and an even smaller proportion has been isolated and characterized at the genetic or functional level. Traditional morphology‐based taxonomy, supported by Sanger sequencing, provided early molecular insights into fungal communities associated with plants (Albrectsen et al. 2010; Sun and Guo 2012; Wijayawardene et al. 2021).

Advances in high‐throughput sequencing, including both second and third‐generation technologies, have revolutionized fungal ecology by enabling culture‐independent assessment of community structure and function (Taylor et al. 2016; Tedersoo et al. 2020; Wijayawardene et al. 2021). Among molecular markers, the nuclear ribosomal internal transcribed spacer (ITS) region is widely recognized as the universal fungal DNA barcode, owing to its high variability, efficient amplification, and extensive representation in the reference databases. The ITS region (~450–750 bp) comprises two spacers, ITS1 and ITS2, separated by the conserved 5.8S gene, flanked by the 18S and 28S ribosomal subunits (Schoch et al. 2012; Nilsson, Anslan, et al. 2019; Siddique et al. 2022). Both ITS1 and ITS2 can recover fungal diversity across multiple taxonomic ranks, although their relative performance varies across lineages, as first demonstrated in comparative analyses of ITS subregions across diverse fungal clades (Seena et al. 2010; Schoch et al. 2012). ITS1 tends to show greater variability in Ascomycota and Basidiomycota, while ITS2 performs more consistently across clades. These lineage‐specific patterns underscore the importance of marker selection when profiling fungal communities using ITS‐based metabarcoding. Short‐read sequencing platforms (e.g., Illumina) typically target either the ITS1 or ITS2 subregion due to read length constraints. Although widely adopted, focusing on a single subregion can limit the discrimination of closely related taxa because of uneven interspecific variability, amplification biases, and insufficient phylogenetic signal. Consequently, many sequences remain unclassified or ambiguously assigned, particularly for taxa with low ITS divergence or poor representation in public databases (Bellemain et al. 2010; Schoch et al. 2012). In contrast, long‐read sequencing technologies such as PacBio can recover full‐length ITS sequences with flanking rRNA genes, potentially enhancing taxonomic assignment depth and providing a more comprehensive view of fungal community structure (Runnel et al. 2022; Mittelstrass et al. 2025). However, the limited availability of full‐length ITS reference databases currently restrict the wider use of long‐read sequencing in fungal community studies.

Another crucial factor influencing the outcome of fungal metabarcoding studies is the primer selection, as different primer pairs targeting the ITS1 or ITS2 subregions exhibit variable coverage and taxonomic bias, ultimately shaping the composition and resolution of recovered communities. Commonly used primers include ITS1F/ITS2 and ITS5/ITS2 for the ITS1 region, and ITS3/ITS4 for the ITS2 region, as well as combinations such as ITS1F/ITS4 to amplify the full‐length ITS region (White et al. 1990; Gardes and Bruns 1993; Toju et al. 2012). While these primers were designed to preferentially target the Kingdom Fungi, their binding sites within conserved ribosomal regions are not exclusive and they can also amplify non‐fungal lineages such as oomycetes (Stramenopiles), which share homologous ribosomal operon structure (Bellemain et al. 2010; Lindahl et al. 2013; Tedersoo et al. 2015). Nevertheless, the complete exclusion of non‐target groups remains challenging, and primer selection should balance broad fungal coverage with minimization of off‐target amplification, depending on the ecological context and study goals (Lindahl et al. 2013; Tedersoo et al. 2015; Nilsson, Anslan, et al. 2019).

Beyond marker selection, fungal community profiling is further influenced by the bioinformatics pipeline. This includes the algorithms used for sequence clustering and taxonomic assignment, as well as the completeness and curation of reference databases. Different algorithms and databases vary in sensitivity, specificity, and taxonomic assignment depth (Pauvert et al. 2019), so these factors can substantially affect estimates of diversity, richness, and relative abundance.

Given the importance of comprehensively characterizing plant‐associated fungal communities, we evaluated the performance of three commonly used ITS markers (ITS1, ITS2, and full‐length ITS, ITSf), using a standardized bioinformatic pipeline (QIIME2 with the scikit‐learn–based classifier and UNITE Eukaryotic database; Bolyen et al. 2019; Nilsson, Larsson, et al. 2019). While previous studies have compared ITS1 and ITS2 in soils and bulk environmental samples (Op De Beeck et al. 2014; Winand et al. 2025), little is known about whether the marker performance varies across different plant tissues. Here, we define “marker performance” as the capacity of ITS markers to retain fungal sequence information after quality filtering and denoising, achieve taxonomic assignment depth, and capture fungal diversity and community compositional patterns. Using a standardized bioinformatic workflow, we compared ITS1, ITS2, and full‐length ITS markers to evaluate how marker choice influences fungal community characterization.

Using DNA from potato ( *Solanum tuberosum* ) leaves and roots, this study provides a comparative evaluation of short‐ and long‐read ITS markers, offering an integrated assessment of how marker choice and sequencing platform jointly influence profiling of fungal and oomycete communities. Comparing ITS markers in potato is particularly relevant because major potato‐associated pathogens include both fungi and oomycetes, such as *Alternaria* and *Phytophthora*, making broad taxonomic recovery important for characterizing plant‐associated microbial communities. We addressed the following questions:

1. How do ITS1, ITS2, and full‐length ITS (ITSf) differ in recovering fungal and oomycete community composition associated with potato leaves and roots?
2. Does marker performance vary between above‐ and below‐ground tissues?
3. Which marker provides the most consistent recovery of taxonomic composition and diversity patterns when analyzed through the same bioinformatic pipeline?

## 2 Materials and Methods

### 2.1 Samples Collection and Experimental Design

Potato plants ( *Solanum tuberosum* L. cv. “Mandel”) were sampled in August 2023, approximately 70 days after planting, at the Röbäcksdalen field station (Umeå, Sweden), as described in Mahawar et al. (2026). The cultivar “Mandel” is a traditional Nordic potato landrace cultivated mainly in northern Scandinavia (Veteläinen et al. 2005) and occurs under several regional names, including “Mandel” (Sweden), “Mandelpotet” or “Gullauge” (Norway), and “Puikula” or “Lapin puikula” (Finland), reflecting diversity within this landrace group (Chrominski et al. 2024).

All samples were immediately preserved on dry ice, transported to the Umeå Plant Science Centre, and stored at −80°C until further processing. Both leaf and root tissues were included to evaluate ITS marker performance across distinct plant‐associated microbial habitats. Because these tissues are characterized by heterogeneous microbial assemblages, pooled composite samples were generated to enable controlled comparative evaluation of ITS marker performance using the same biological material across sequencing approaches. Eight samples from each tissue type (leaf and root), previously described in Mahawar et al. (2026), were randomly selected and combined prior to DNA extraction. The resulting composite samples were used for comparative sequencing and downstream benchmarking of ITS1, ITS2, and full‐length ITS (ITSf) markers.

### 2.2 DNA Extraction, PCR Amplification, Library Preparation, and Sequencing

Frozen tissues were lyophilized prior to processing. For each tissue type, approximately 0.5 g of tissue from each selected sample was combined to generate pooled composite material for comparative ITS marker evaluation. Composite samples were homogenized into a fine powder using a sterile mortar and pestle prior to DNA extraction and amplicon sequencing.

Genomic DNA was extracted from 50 mg of powdered tissue per composite sample using the DNeasy Plant Pro Kit (QIAGEN, Hilden, Germany) according to the manufacturer's instructions. Extractions were performed in technical duplicates. DNA concentration and purity were assessed using a Qubit 2.0 Fluorometer with the Qubit dsDNA HS Assay Kit (Invitrogen, Thermo Fisher Scientific Inc., Waltham, MA, USA) and a NanoDrop spectrophotometer (Thermo Fisher Scientific Inc., Waltham, MA, USA). Samples were required to meet the sequencing facility (Novogene) quality criteria for PCR amplification and library preparation: DNA concentration ≥ 10 ng μL^−1^, total DNA ≥ 200 ng, volume ≥ 20 μL, and purity (OD260/280) between 1.8 and 2.0. For each composite sample, the extraction with the highest DNA quality was selected for downstream processing. If neither extraction replicate met the required thresholds, DNA was re‐extracted from the corresponding pooled material.

Each DNA extract was divided into three aliquots prior to shipment because separate reactions were required for amplification of the three target regions analyzed in this study (ITS1, ITS2, and full‐length ITS [ITSf]). One aliquot per target region was sent to Novogene (Cambridge, UK) for PCR amplification, library preparation, and sequencing following the company's standard protocols.

Region‐specific primers were used for each target region: ITS1: ITS5 (5′‐GGAAGTAAAAGTCGTAACAAGG‐3′)/ITS2 (5′‐GCTGCGTTCTTCATCGATGC‐3′); ITS2: ITS3 (5′‐GCATCGATGAAGAACGCAGC‐3′)/ITS4 (5′‐TCCTCCGCTTATTGATATGC‐3′); full‐length ITSf: ITS9MUNngs (5′‐TACACACCGCCCGTCG‐3′)/ITS4ngsUni (5′‐CCTSCSCTTANTDATATGC‐3′).

PCR amplification was carried out using 15 μL Phusion High‐Fidelity PCR Master Mix (New England Biolabs) with approximately 10 ng of template DNA and 0.2 μM of each forward and reverse primer. PCR cycling conditions consisted of an initial denaturation at 98°C for 1 min, followed by 30 cycles of denaturation at 98°C for 10 s, annealing at 50°C for 30 s, and extension at 72°C for 30 s, with a final extension at 72°C for 5 min. PCR products were verified on 2% agarose gels and purified using magnetic beads. Equimolar amounts of PCR products were pooled, end‐repaired, A‐tailed, and ligated with sequencing adapters (Illumina adapters for Illumina libraries and SMRTbell adapters for PacBio libraries). Libraries were quantified using Qubit and size distributions were assessed using a Bioanalyzer (Agilent Technologies, Santa Clara, California, USA).

ITS1 and ITS2 amplicons were sequenced on an Illumina NovaSeq 6000 platform (paired‐end, 2 × 250 bp), whereas full‐length ITS (ITSf) amplicons were sequenced using PacBio Revio HiFi reads. All sequencing data have been deposited in the NCBI Sequence Read Archive (BioProject accession PRJNA1330153).

### 2.3 Bioinformatic and Statistical Analysis

Bioinformatics analyses were performed using the Quantitative Insight into Microbial Ecology 2 (QIIME2, v.2024.10), following the pipeline described by Turco et al. (2024), Cardacino et al. (2025), Giubilei et al. (2026). Illumina paired‐end reads (2 × 250 bp) were merged and quality‐filtered by Novogene using their standard amplicon pipeline. The resulting merged Illumina reads and filtered full‐length ITS (ITSf) PacBio HiFi reads were imported into the QIIME2 environment for downstream analyses. After demultiplexing, chimera filtering, and denoising with the Divisive Amplicon Denoising Algorithm 2 (DADA2), taxonomic classification was performed using a scikit‐learn–based classifier and UNITE v10 release 2024‐04‐21 as a reference database (Abarenkov et al. 2024). Performance metrics for ITS1, ITS2, and ITSf were evaluated according to retained read proportions after quality filtering and denoising, together with taxonomic assignment rates. Efficiency was calculated as the proportion of retained reads after quality filtering and denoising relative to the number of raw reads. Taxonomic assignment rates were evaluated across multiple taxonomic ranks, including family, genus, and species levels, to compare the relative classification depth achieved by each ITS marker. However, downstream ecological inference and community composition analyses were conducted primarily at the genus level because of the lower confidence and consistency generally associated with species‐level assignments in ITS‐based metabarcoding datasets.

Features table, taxonomy table, representative sequences and a metadata file with sample description were then imported into the R environment (v4.2.3) for further processing using *phyloseq* (v1.52) and *vegan* (v2.7‐1) packages. Although the study primarily focused on fungal communities, oomycete ASVs were retained in downstream analyses because ITS primers also amplify Stramenopila taxa and because oomycetes such as *Phytophthora* are ecologically relevant plant‐associated pathogens in potato. Prior to alpha‐ and beta‐diversity analyses, plant‐derived ASVs were removed from the dataset, while fungal and oomycete ASVs were retained because of their ecological relevance in the potato pathosystem. For alpha‐ and beta‐diversity analyses, ASV tables were rarefied to the minimum sample size depth to control for library size variation (42 reads for leaves and 299 reads for roots, respectively). The low rarefaction depth observed in leaf samples primarily reflected extensive host‐derived amplification. For compositional summaries and relative abundance visualization, ASV counts were transformed to relative abundances. Alpha diversity richness and evenness was assessed using the Shannon and Pielou indexes, and the statistical significance evaluated with a Kruskal‐Wallis test. Beta diversity among samples was analyzed using non‐metric multidimensional scaling (NMDS) and Principal Coordinates Analysis (PCoA) based on Bray–Curtis dissimilarity matrices. The effects of ITS region and tissue type (leaves and roots) on fungal and oomycete community composition were tested using permutational multivariate analysis of variance (PERMANOVA; 999 permutations). Because PERMANOVA can be sensitive to differences in multivariate dispersion among groups, homogeneity of dispersion was additionally assessed using PERMDISP analysis (*betadisper* and *permutest* functions in the vegan package; 999 permutations). Because biological material was pooled prior to sequencing, the resulting statistical analyses should be interpreted primarily as exploratory comparisons of marker‐associated compositional patterns rather than population‐level ecological inference. The relative abundance of fungal and oomycete taxa was visualized at the genus level using the *ggplot2* package, while Venn diagrams were plotted to visualize shared genera among ITS markers. For visualization of dominant community patterns, ASVs were agglomerated at the genus level using the *tax_glom* function in *phyloseq*. Replicate pairs were merged by summing counts, transformed to relative abundances, and the 30 most abundant genera were selected based on mean relative abundance across samples. Relative abundance patterns were visualized using row‐wise *Z*‐score normalization with the *ComplexHeatmap* package in R. Putative functional guild assignments were inferred using the *fungaltraits* package (v0.0.3) in R, which integrates both FunGuild and FunFun databases (Põlme et al. 2020).

## 3 Results

### 3.1 Sequencing and DADA2 Denoise Output

The number of input reads before and after demultiplexing, denoising, and chimera filtering with DADA2, along with the total number of Amplicon Sequence Variants (ASVs) per sample, is summarized in Table S1. Across all markers and tissues, the majority of reads were assigned to plant, indicating substantial co‐amplification of host DNA (Figure S1B). In leaves, plant reads dominated the datasets for all markers, reaching ~298, ~317 and ~90k reads for ITS1, ITS2 and ITSf respectively, whereas reads assigned to Fungi + Stramenopila were much lower (17,473 for ITS1, 555 for ITS2 and 5574 for ITSf). A similar pattern was observed in roots, with plant reads exceeding 300k for ITS1 and ITS2 and ~95k for ITSf, while fungal and stramenopile reads reached 19,848 (ITS1), 12,912 (ITS2) and 1788 (ITSf) (Table S1). Consequently, relative amplification efficiency was strongly biased toward plant DNA for all markers, representing between ~94%–99% of total reads (Figure S1B). When considering only reads belonging to Fungi and Stramenopila, ITS1 consistently recovered the highest number of classified reads and showed high assignment rates across taxonomic levels (Figure S1C). In leaves, ITS1 recovered 17,473 reads (Table S1) belonging to Fungi and Stramenopila, with most of them successfully classified at deeper taxonomic levels (17,391 at family, 17,386 at genus and 17,341 at species). In roots, ITS1 recovered 19,848 fungal and stramenopile reads (Table S1), of which 19,655 and 19,462 were classified at the family and genus levels, respectively, while 11,444 reads were resolved to species level. ITSf showed intermediate performance (~5.5k reads at family level and slightly fewer at genus and species levels). In roots, ITS1 again produced the largest number of classified reads (~19.7k at family level and ~11.4k at species level), followed by ITS2 (~12.3k family reads but only ~4.7k classified to species) and ITSf (~1.8k family reads and ~1.6k species reads) (Figure S1C). Although assignment rates were evaluated through species level for comparative benchmarking purposes, downstream ecological analyses were performed primarily at the genus level because of the lower confidence associated with species‐level ITS classifications. Overall, ITS1 provided the highest recovery of fungal and oomycete reads together with the greatest depth of taxonomic assignment, whereas ITS2 appeared more strongly affected by host‐derived amplification, particularly in leaf samples.

**FIGURE 1:**
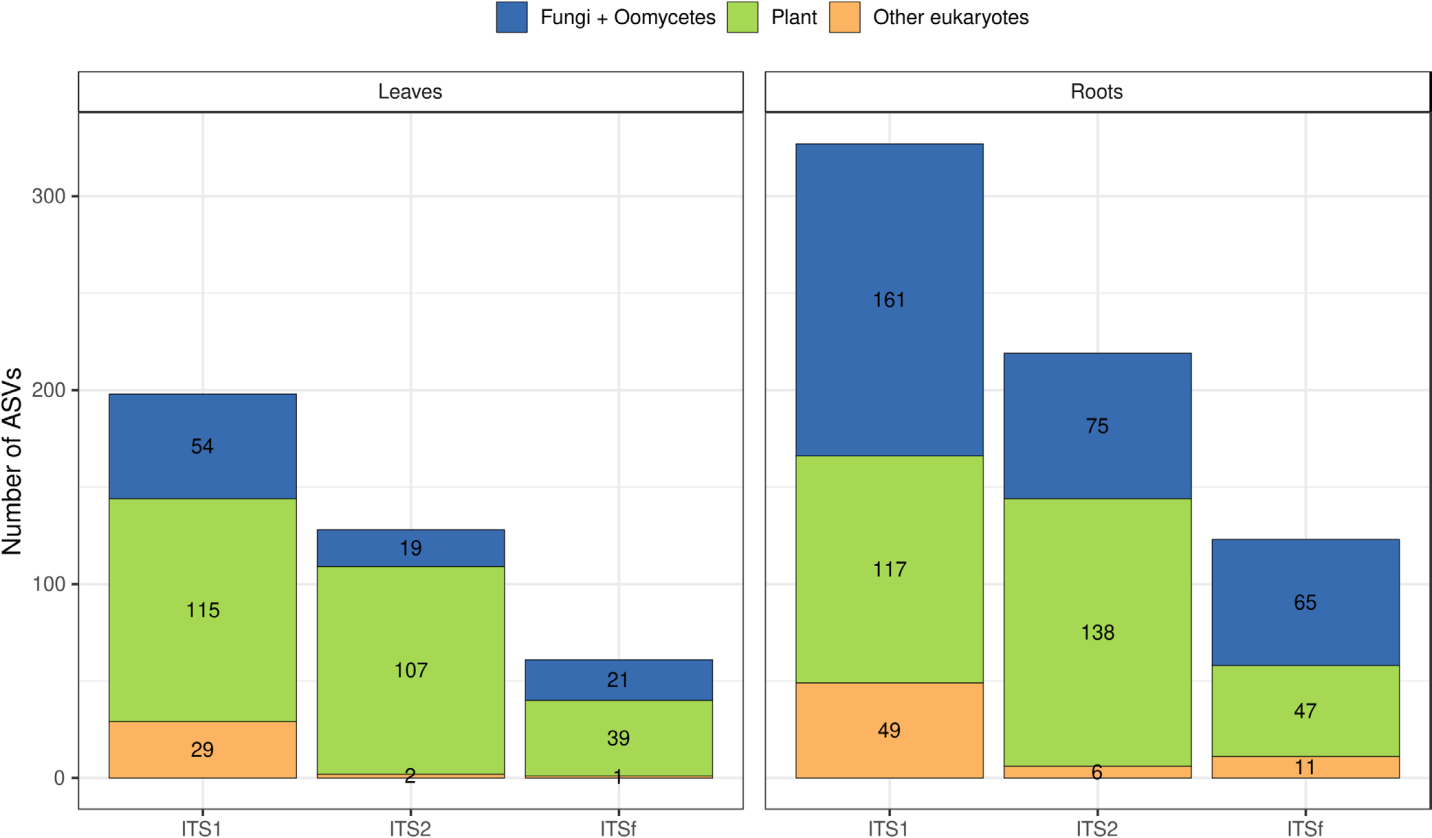
Number of ASVs assigned to fungi and oomycetes (blue), plants (green), or other sources (orange) across markers (ITS1, ITS2, ITSf) and tissues (leaves, roots).

The number of ASVs detected varied among markers and tissues (Figure 1). In leaves, plant‐derived ASVs represented the largest fraction across all markers, with 115 and 107 ASVs detected with ITS1 and ITS2, respectively, and 39 with ITSf. Fungal and oomycete ASVs were less abundant in leaves, with 54 detected using ITS1, 19 using ITS2, and 21 using ITSf. In contrast, root samples showed a higher number of fungal and oomycete ASVs, particularly with ITS1 (161 ASVs), followed by ITS2 (75 ASVs) and ITSf (654 ASVs). Plant‐derived ASVs were also abundant in roots, with the highest number detected using ITS2 (138 ASVs), compared with 117 and 47 ASVs detected using ITS1 and ITSf, respectively. ASVs assigned to other eukaryotes were detected at relatively low numbers across markers and tissues.

### 3.2 Taxonomic Classification

**FIGURE 2:**
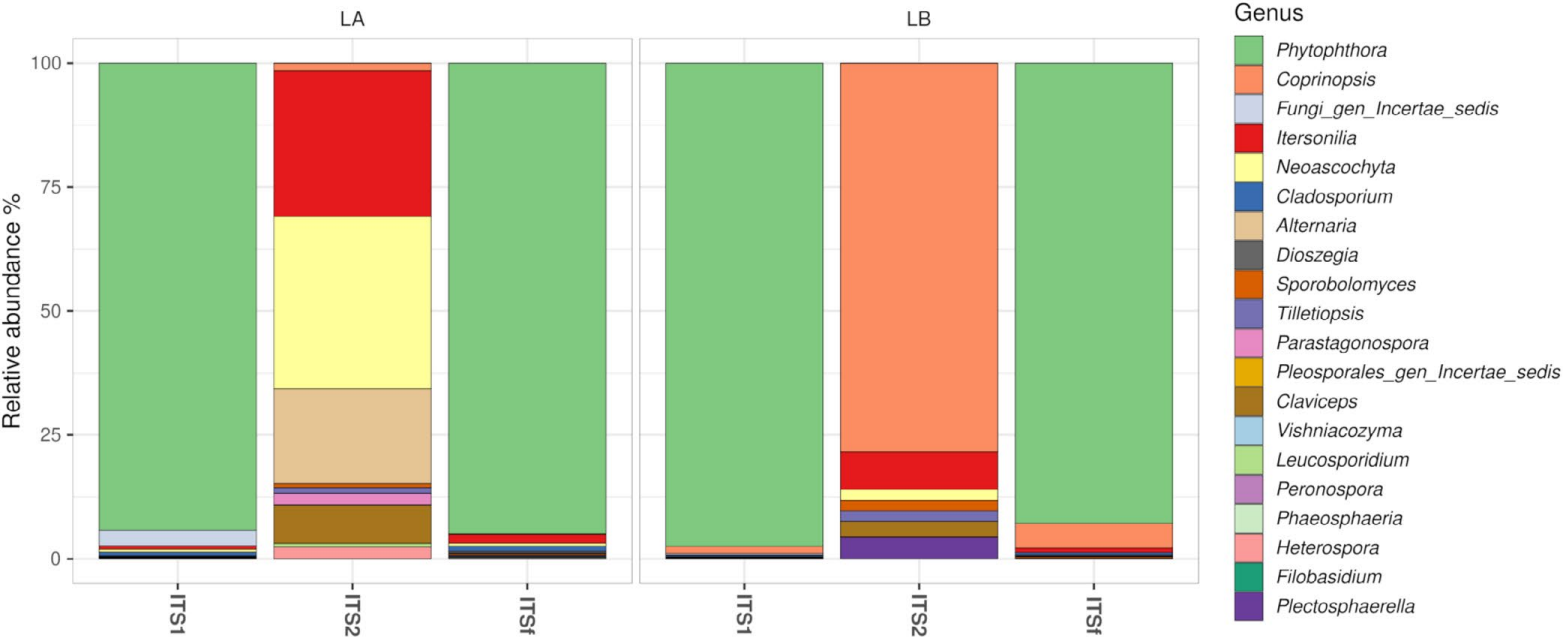
Relative abundance of genera detected in potato leaves with three ITS markers (ITS1, ITS2, and full‐length ITS).

Besides fungi, non‐fungal eukaryotes (e.g., Viridiplantae, Stramenopila, Metazoa, Rhizaria, Alveolata) were co‐amplified to varying degrees, reflecting the broad eukaryotic coverage of ITS priming sites (Figure S2 and Table S2). In leaves (LA and LB), plant‐derived sequences accounted for the vast majority of reads, exceeding 93%–99% across all markers, with the highest proportions observed for ITS2 (> 99%). Fungal sequences assigned to Ascomycota were detected at very low levels in leaves (< 0.3%), whereas Oomycota, detected mainly with ITS1 and ITSf (4%–6%), were not captured by ITS2. In roots (RA and RB), Ascomycota showed higher relative abundances, particularly with ITS1 (8.3% in RA and 2.5% in RB) and ITS2 (5.3% in RA and 2.7% in RB), while remaining low with ITSf (< 1%). Oomycota were consistently rare in roots (< 1%), and were not detected with ITS2. Metazoa, Rhizaria and Alveolata are included within < 1%. After removal of plant‐derived ASVs, the 20 most abundant genera revealed clear differences among ITS regions in leaf communities (Figure 2 and Table S3). In both leaf groups, ITS1 and ITSf libraries were overwhelmingly dominated by *Phytophthora*, which accounted for an average of 94.3% (LA) and 97.5% (LB) of the ITS1 community, and 94.9% (LA) and 92.8% (LB) of the ITSf community. In contrast, ITS2 recovered a markedly more diverse assemblage and showed clear differences between LA and LB. In LA, ITS2 libraries were mainly composed of *Neoascochyta* (34.8%), *Itersonilia* (29.4%), and *Alternaria* (19.1%), with smaller contributions from *Claviceps* (7.7%), *Heterospora* (2.4%), and *Parastagonospora* (2.4%). In LB, ITS2 was strongly dominated by *Coprinopsis* (78.4%), followed by *Itersonilia* (7.6%), *Plectosphaerella* (4.3%), and *Claviceps* (3.3%), whereas *Neoascochyta*, *Tilletiopsis*, and *Sporobolomyces* each contributed about 2.1%. Overall, ITS1 and ITSf produced highly similar profiles dominated by *Phytophthora*, whereas ITS2 recovered a broader and more heterogeneous set of leaf‐associated fungi.

**FIGURE 3:**
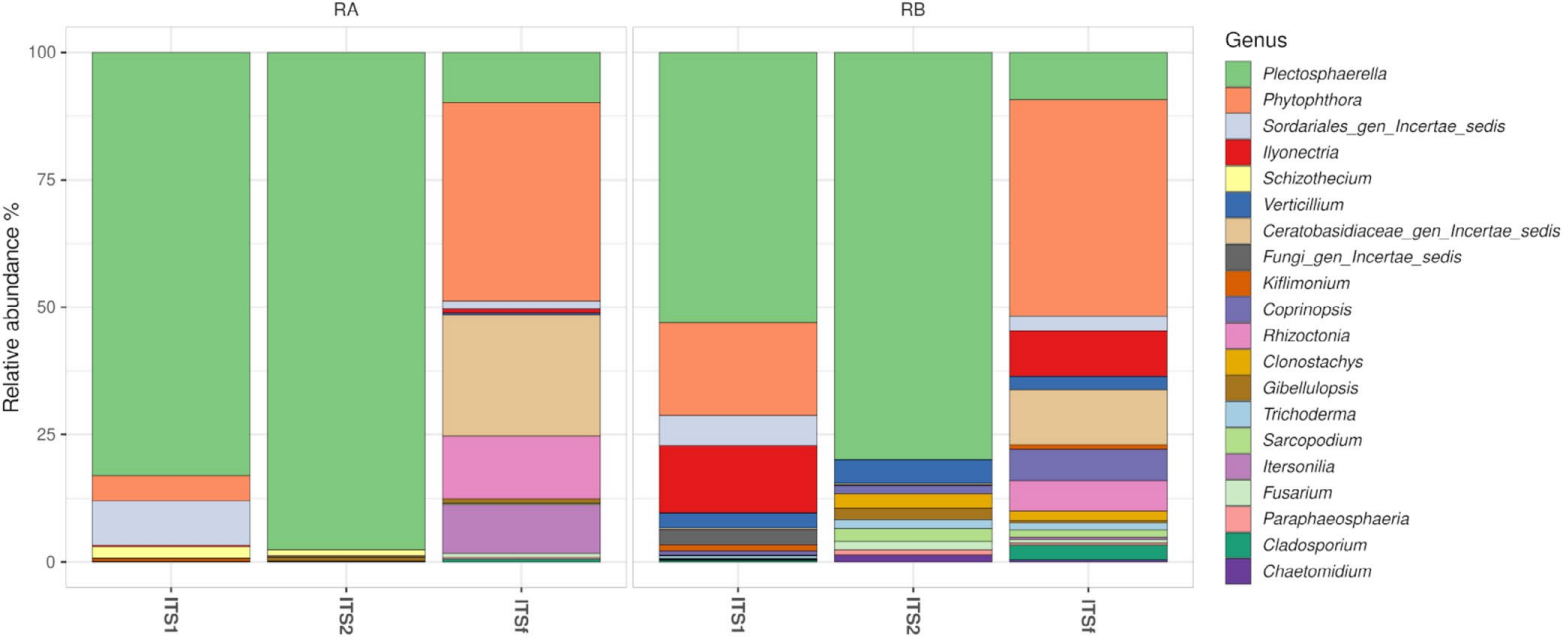
Relative abundance of genera detected in potato roots with three ITS markers (ITS1, ITS2, and full‐length ITS).

In root samples, fungal communities were strongly dominated by a limited number of taxa, with clear differences among markers (Figure 3 and Table S3). Across ITS1 and ITS2 libraries, the genus *Plectosphaerella* represented the most abundant taxon, accounting for up to 96.3%–98.9% of reads in RA samples and 76.8%–83.1% in RB samples. In contrast, ITSf libraries were less dominated by a single genus and showed a higher contribution of multiple taxa. In these samples, *Phytophthora* was among the most abundant taxa, reaching 51.7% in RA and 41.8%–43.2% in RB samples. Other relevant taxa detected in ITSf included *Ceratobasidiaceae* (up to 24.6%), *Rhizoctonia* (up to 14.6%), and *Itersonilia* (up to 14.2%). Additional genera such as *Ilyonectria*, *Verticillium*, and *Coprinopsis* were also detected, generally at lower relative abundances (< 15%). Overall, ITS1 and ITS2 libraries were largely dominated by *Plectosphaerella*, whereas ITSf recovered a broader assemblage including both fungal taxa and oomycetes such as *Phytophthora*.

### 3.3 Diversity Indices of Fungal Communities

**FIGURE 4:**
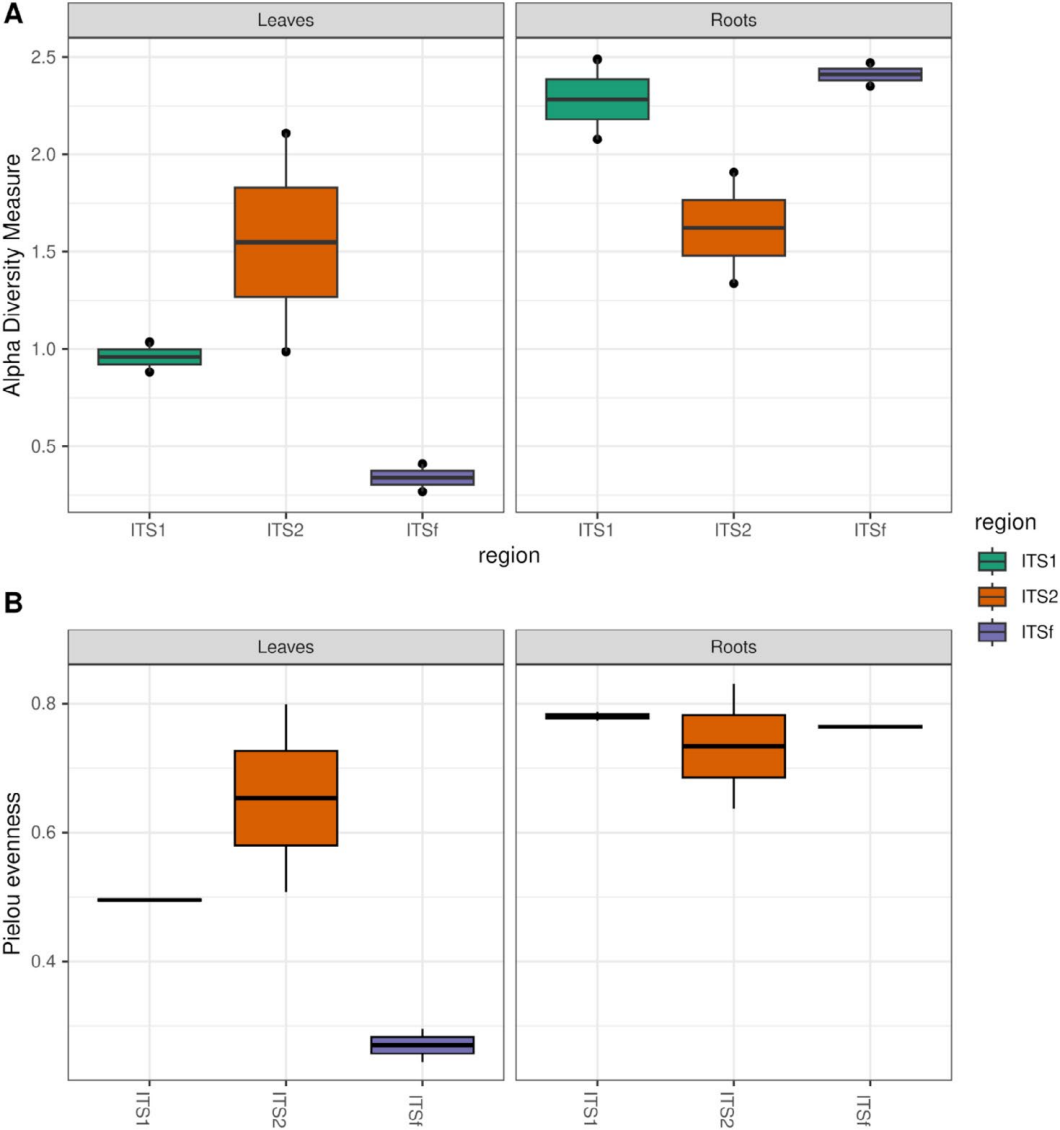
Alpha‐diversity metrics of fungal communities comparing ITS markers in two potato tissues. Boxplots highlight strong marker‐ and tissue‐specific differences in fungal diversity and community structure.

Alpha‐diversity patterns differed between tissues and among ITS markers (Figure 4). In leaf samples, ITS2 recovered the most diverse and even fungal communities, with Shannon diversity values between 1.3–1.8 and Pielou's evenness between 0.6–0.8. In contrast, ITS1 yielded intermediate diversity in leaves (Shannon: 0.9–1.0, evenness: 0.5), whereas ITSf consistently showed the lowest diversity and evenness (Shannon: 0.3–0.4, evenness: 0.25–0.30), indicating communities strongly dominated by a few taxa. These patterns are consistent with the taxonomic composition results, in which ITS2 recovered a broader range of leaf‐associated fungi, whereas ITS1 and especially ITSf libraries were largely dominated by *Phytophthora*.

In the roots, the pattern differed markedly. Both ITS1 and ITSf showed higher alpha diversity than ITS2, with ITSf displaying the highest Shannon values overall (up to 2.4) and high evenness (up to 0.76). ITS1 also recovered highly diverse and even root communities, with Shannon values around 2.2–2.4 and evenness close to 0.78. By contrast, ITS2 showed lower diversity in roots (Shannon: 1.5–1.8) and slightly lower evenness (0.7–0.8), reflecting communities more strongly dominated by *Plectosphaerella*.

Statistical analysis confirmed that tissue type significantly influenced Shannon diversity (Kruskal–Wallis test, *χ* ^2^ = 5.77, df = 1, *p* = 0.016), with roots exhibiting overall higher diversity than leaves. In contrast, no significant differences in Shannon diversity were detected among ITS regions (Kruskal–Wallis test, *χ* ^2^ = 0.15, df = 2, *p* = 0.926), indicating that variation in diversity was primarily associated with tissue type rather than marker region.

**FIGURE 5:**
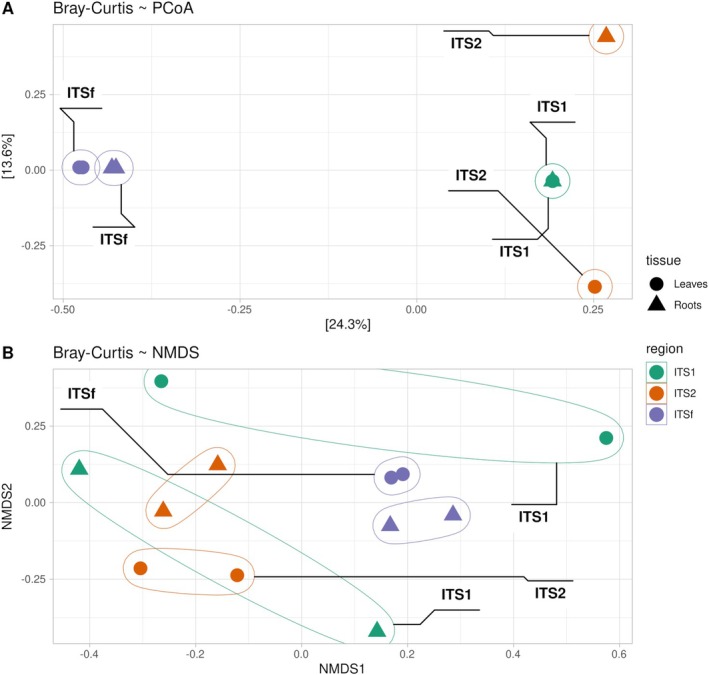
Principal Coordinates Analysis (PCoA) of the beta‐diversity based on Bray–Curtis dissimilarities in leaves (A) and roots (B) tissues. Community clustering was mainly influenced by marker choice (ITS1 = green, ITS2 = orange, ITSf = purple).

Consistent with the alpha‐diversity patterns, which revealed strong marker‐ and tissue‐dependent differences in diversity, beta‐diversity analyses based on Bray–Curtis dissimilarity showed that fungal community composition was primarily structured by ITS marker choice (Figure 5). Non‐metric multidimensional scaling (NMDS) showed substantial overlap among samples, with no clear separation between groups, although some tendency toward marker‐related structuring could be observed. Differences between leaf and root samples were also not clearly resolved in ordination space. PERMANOVA analysis confirmed that the ITS region was the main factor explaining community variation (*R* ^2^ = 0.36, *F* = 2.66, *p* = 0.001). In contrast, tissue type explained a smaller proportion of the variance (*R* ^2^ = 0.10) and was only marginally non‐significant (*p* = 0.055). To assess whether PERMANOVA results were influenced by heterogeneity in multivariate dispersion, PERMDISP analysis was performed. Significant differences in dispersion were detected among ITS regions (*p* = 0.002), whereas no significant dispersion differences were observed between tissue types (*p* = 0.907). Therefore, the effect associated with ITS marker choice should be interpreted with caution, as part of the observed separation may reflect differences in dispersion among groups rather than centroid separation alone. Overall, these results indicate that marker choice strongly influences both alpha‐ and beta‐diversity patterns, whereas differences between tissues contribute more modestly to community structuring.

### 3.4 Performance of ITS Markers in Detecting Unique and Overlapping Genera

**FIGURE 6:**
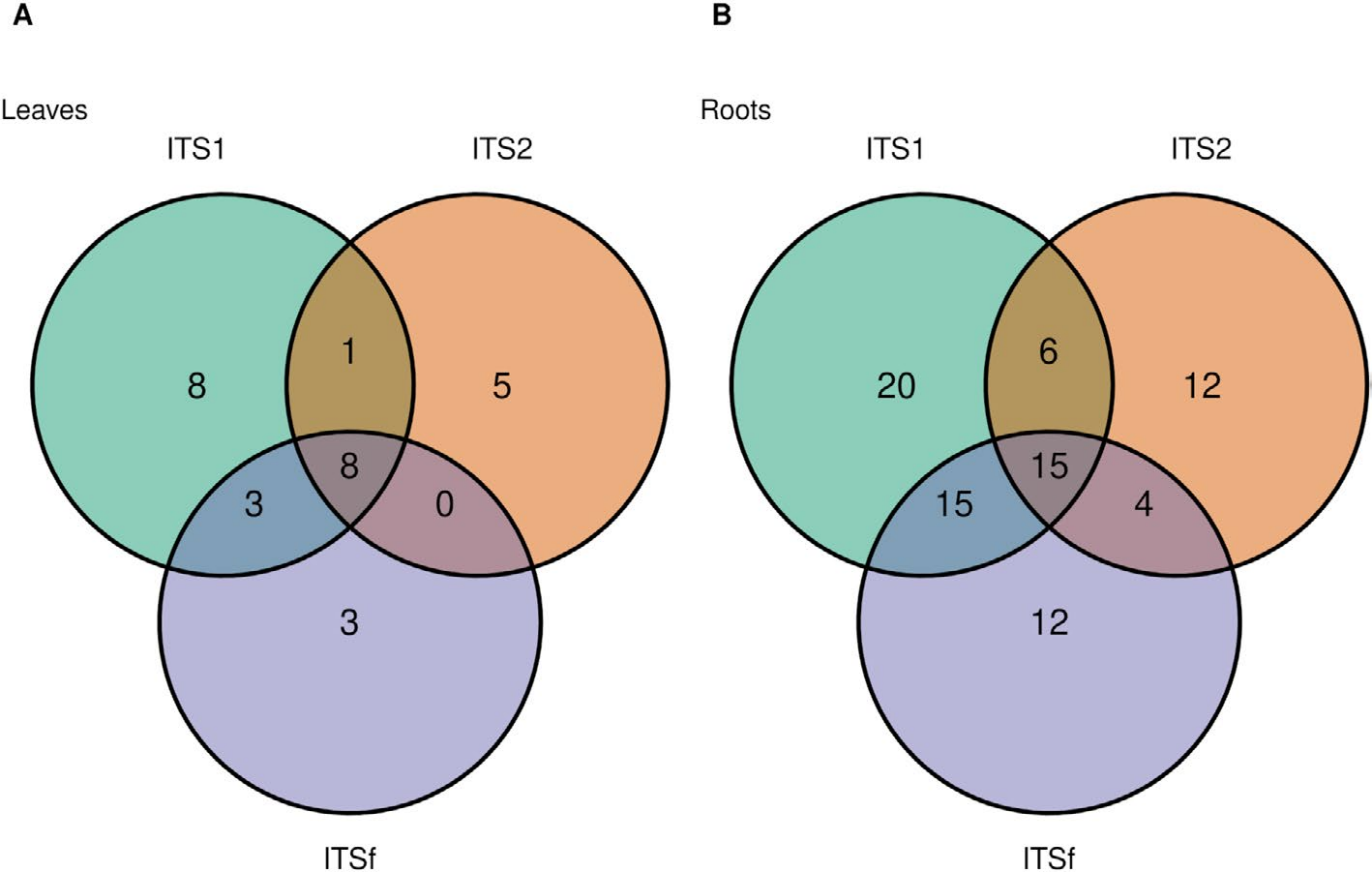
Venn diagrams showing the overlap and unique distribution of microbial genera detected across three ITS markers representing two potato tissues. Numbers indicate the genera shared among or unique to each marker, illustrating strong marker‐specific biases in microbial community detection.

Venn diagram analysis revealed limited overlap in fungal genera detected by the three ITS markers, with pronounced marker‐specific patterns in both leaf and root tissues (Figure 6 and Table S4). In leaves, eight genera were consistently detected across all markers, forming a shared core composed of *Alternaria, Coprinopsis, Itersonilia, Leucosporidium, Neoascochyta, Parastagonospora, Sporobolomyces*, and *Tilletiopsis*. Beyond this core, overlap among markers was limited. ITS1 and ITSf jointly detected *Cladosporium, Dioszegia*, and *Phytophthora*, whereas ITS1 and ITS2 shared only *Claviceps*, and no genera were exclusively shared between ITS2 and ITSf. Each marker also recovered distinct fungal lineages. ITS1 uniquely detected genera such as *Amarenographium, Harzia, Leptobacillium, Peronospora*, and *Selenophoma*, along with unclassified taxa (*Fungi_gen_Incertae_sedis* and *Pleosporales_gen_Incertae_sedis*). ITS2 uniquely recovered *Erysiphe, Fusarium, Heterospora, Plectosphaerella*, and *Septoriella*, while ITSf specifically detected yeast‐like and filamentous taxa including *Filobasidium, Phaeosphaeria*, and *Rhodotorula*.

In roots, overlap among markers was greater but still accompanied by substantial marker‐specific detection. Fifteen genera were shared across all three markers, including ecologically relevant taxa such as *Coprinopsis, Fusarium, Mortierella, Penicillium, Trichoderma*, and *Verticillium*, along with *Helminthosporium, Itersonilia*, and *Plectosphaerella*. Pairwise overlaps were particularly strong between ITS1 and ITSf, which jointly detected genera such as *Cladosporium, Dioszegia, Ilyonectria, Phytophthora, Rhizoctonia*, and *Sporothrix*. Additional overlap between ITS1 and ITS2 included *Exophiala, Schizothecium*, and *Torula*, while ITS2 and ITSf shared fewer taxa, such as *Clonostachys* and *Gibellulopsis*. Despite this overlap, each marker revealed a large number of unique genera. ITS1 uniquely detected diverse taxa including *Acremonium, Apiospora, Hymenoscyphus, Paecilomyces, Parasola*, and *Septoria*, ITS2 uniquely recovered genera such as *Agrocybe, Dactylonectria, Neosetophoma*, and *Sarocladium*, and ITSf uniquely identified taxa including *Mucor, Naganishia, Linnemannia*, and *Spizellomyces*.

### 3.5 Relative Abundance Patterns of Dominant Genera

**FIGURE 7:**
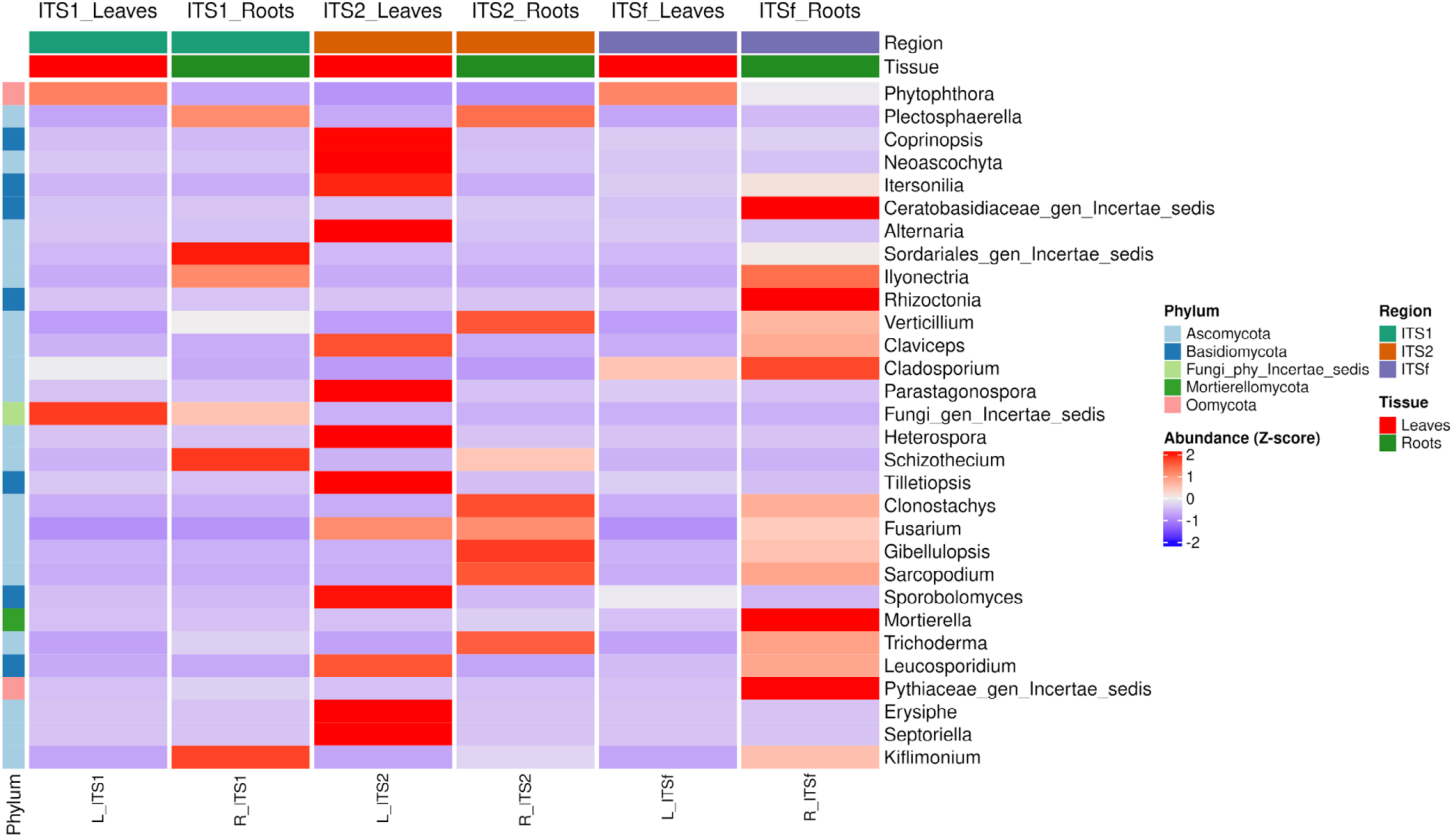
Heatmap showing relative abundance patterns of the 30 most abundant fungal and oomycete genera across ITS markers and tissue types. Values represent row‐wise *Z*‐score normalized relative abundances calculated after genus‐level agglomeration and merging of replicate pairs. Row annotations indicate fungal phyla, whereas column annotations indicate ITS marker and tissue type.

A genus‐level heatmap of relative abundance patterns across ITS markers and tissue types revealed strong marker‐ and tissue‐associated differences in community composition (Figure 7). The heatmap was based on the 30 most abundant genera and visualized using row‐wise *Z*‐score normalization of relative abundance values. Taxa exhibited distinct abundance patterns depending on the amplified region (ITS1, ITS2, ITSf) and the biological context (leaf vs. root).

In leaf tissues, ITS2 showed the highest relative abundance across a wide range of genera, including *Coprinopsis, Neoascochyta, Itersonilia, Alternaria, Claviceps, Parastagonospora, Heterospora, Tilletiopsis, Sporobolomyces, Erysiphe*, and *Septoriella*. In contrast, ITS1 leaves were primarily characterized by higher relative abundance of *Phytophthora* and *Fungi_gen_Incertae_sedis*, while ITSf leaves displayed weaker and more limited representation patterns, with only a few taxa such as *Cladosporium* showing relatively higher values. These patterns suggest that ITS2 recovered a broader range of foliar‐associated taxa, consistent with previous observations.

In root tissues, relatively abundant patterns were broader and more evenly distributed across markers. ITSf roots were associated with increased relative abundance of *Ceratobasidiaceae_gen_Incertae_sedis, Ilyonectria, Rhizoctonia, Mortierella*, and *Pythiaceae_gen_Incertae_sedis*, along with moderate representation of *Clonostachys, Fusarium, Gibellulopsis, Sarcopodium*, and *Trichoderma*. ITS2 roots also showed higher relative abundance of several root‐associated genera, including *Verticillium, Clonostachys, Fusarium, Gibellulopsis, Sarcopodium*, and *Trichoderma*, indicating its ability to resolve complex soil‐borne fungal communities. In contrast, ITS1 roots were characterized by comparatively high abundance of *Plectosphaerella, Sordariales_gen_Incertae_sedis, Schizothecium*, and *Kiflimonium*.

The heatmap also highlights the detection of ecologically relevant or niche‐associated taxa, such as *Ceratobasidiaceae_gen_Incertae_sedis, Rhizoctonia, Claviceps, Sarcopodium*, and *Peziza*, which were preferentially detected by ITSf or dominant in root compartments. This suggests that certain functional groups or less‐characterized fungal clades may be underrepresented depending on marker choice, emphasizing the value of multi‐marker approaches for comprehensive community profiling. Basidiomycota and occasional non‐fungal groups appeared sporadically, particularly in ITSf root datasets, likely reflecting off‐target amplification or background environmental DNA.

### 3.6 Functional Classification

**FIGURE 8:**
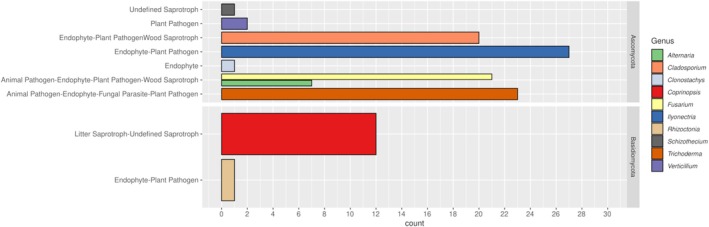
Functional guild distribution of fungal genera based on FUNGuild annotation. Genera are grouped by higher taxonomic clade (Ascomycota and Basidiomycota), and bars represent the number of occurrences assigned to each guild category. Colors indicate individual genera. Many taxa were assigned to multiple guilds, resulting in complex, overlapping functional classifications.

Putative functional guild assignments inferred using FUNGuild suggested that most detected genera exhibited multi‐functional ecological roles, with a strong dominance of mixed guild assignments, particularly within Ascomycota (Figure 8). The majority of taxa were classified into combined categories such as endophyte–plant pathogen and broader multi‐guild groups, reflecting the ecological versatility of genera such as *Fusarium, Trichoderma*, and *Ilyonectria*. Among these, *Ilyonectria* showed the highest representation within endophyte–plant pathogen categories, while *Fusarium* and *Trichoderma* were associated with highly complex guild combinations including pathogenic, saprotrophic, and parasitic traits. In contrast, Basidiomycota exhibited a simpler functional structure, largely dominated by saprotrophic taxa such as *Coprinopsis*, alongside the plant pathogen *Rhizoctonia*. The presence of taxa assigned to multiple ecological roles highlights the limitations of genus‐level functional annotation and underscores the broad ecological plasticity of many fungal lineages. Overall, these results indicate that functional inference is strongly influenced by taxonomic composition and that many dominant genera cannot be unambiguously assigned to a single ecological guild.

## 4 Discussion

The present study demonstrates that ITS marker selection substantially influences observed fungal community composition and downstream ecological interpretation. Across potato leaves and roots, marker choice was associated with a greater proportion of observed variation in community composition than tissue type, indicating that marker selection is a major factor associated with metabarcoding results. Our comparison revealed clear marker‐specific strengths and limitations, highlighting trade‐offs between amplification efficiency, read recovery, and taxonomic assignment depth. Importantly, marker performance was strongly tissue‐dependent: ITS2 generally recovered more diverse and even profiles in leaf tissues, whereas full‐length ITS (ITSf) recovered comparatively more even and compositionally broader root‐associated communities, as reflected by higher Shannon diversity and evenness values, despite substantially lower sequencing depth compared to short‐read Illumina approaches. In contrast, ITS1 showed high amplification efficiency but generated strongly skewed community structures, largely driven by the overrepresentation of oomycetes such as *Phytophthora*. While this bias limits its suitability for comprehensive community profiling, it may represent a valuable tool in pathosystems such as potato, where *Phytophthora* species are among the most economically destructive pathogens affecting *Solanum tuberosum* worldwide (Coomber et al. 2023).

The differential performance of ITS markers reflects both intrinsic properties of the targeted regions and their interaction with tissue‐specific characteristics. ITS2 consistently provided the most informative and diverse profiles in leaf samples, as reflected by higher Shannon diversity (approximately 1.3–1.8) and evenness values (Pielou index ~0.6–0.8) compared to the other markers. Although differences in alpha diversity among ITS regions were not statistically significant overall (Kruskal–Wallis test, *χ* ^2^ = 0.15, df = 2, *p* = 0.926), these consistent patterns across leaf samples indicate that ITS2 appeared to recover a broader and more evenly distributed fungal profile in the phyllosphere, consistent with the taxonomic composition results. This apparent discrepancy between alpha‐ and beta‐diversity results reflects the fact that diversity metrics and community composition capture different aspects of microbial communities, with the former describing richness and evenness, and the latter reflecting differences in taxonomic composition among samples.

Because each ITS marker recovered partially distinct subsets of the detected community, the resulting profiles should be interpreted as complementary rather than definitive representations of the underlying plant‐associated microbiome. These findings are consistent with previous studies reporting that ITS2 provides broader taxonomic assignment depth across diverse fungal clades (Blaalid et al. 2013; Nilsson, Larsson, et al. 2019; Winand et al. 2025). The shorter amplicon length and extensive representation in reference databases likely explain its superior performance in leaf tissue, where fungal biomass is relatively low and host contamination is high.

By contrast, full‐length ITS (ITSf) performed better in root samples, recovering more diverse and balanced fungal communities, as reflected by higher Shannon diversity and evenness values compared to the other markers, and generating more evenly distributed community structures compared to short‐read markers. Beta‐diversity analyses indicated that ITS marker choice was associated with substantial variation in observed root‐associated community composition (PERMANOVA: *R* ^2^ = 0.36, *p* = 0.001), although interpretation should be made cautiously because PERMDISP analysis detected significant differences in multivariate dispersion among markers. ITSf recovered a broader range of ecologically diverse taxa relative to the short‐read markers (Tedersoo et al. 2018), suggesting that this marker may provide broader coverage of root‐associated communities, which are generally more complex and heterogeneous than those found in leaves. The ability of ITSf to span both ITS1 and ITS2, together with parts of the flanking rRNA genes, may help reduce misclassification of closely related lineages and increase the detection of rare or underrepresented taxa (Tedersoo et al. 2018; Runnel et al. 2022).

ITS1, while showing high amplification efficiency and recovering a large number of fungal and oomycete ASVs, produced strongly skewed community profiles across both tissues. This indicates that high read recovery does not necessarily translate into a representative view of community composition, as ITS1‐derived communities were often dominated by a limited number of taxa, particularly oomycetes such as *Phytophthora*. Despite relatively high richness, ITS1‐derived communities exhibited low evenness, with a disproportionate contribution of a few dominant lineages, especially in leaf samples. Such skewed distributions suggest that ITS1 is more prone to amplification biases, likely related to primer specificity and differential template affinity. These limitations reflect primer mismatches, intragenomic heterogeneity, and limited ITS1 representation in databases (Bellemain et al. 2010; Blaalid et al. 2013; Mbareche et al. 2021). However, this apparent limitation may also represent a context‐dependent advantage in pathosystems such as potato, where the detection of key pathogenic taxa is of primary importance. In particular, the consistent detection of *Phytophthora*, a major pathogen of potato responsible for severe yield losses worldwide, highlights the potential of ITS1 as a sensitive marker for pathogen surveillance in plant‐associated microbiome studies. The observed marker performance was strongly tissue‐dependent, with leaf‐associated communities more effectively resolved by ITS2 and root‐associated communities more comprehensively captured by ITSf. These differences likely reflect variation in fungal biomass and community complexity between tissues. Technical factors such as amplicon length and host DNA interference may also contribute to the observed marker‐specific patterns. The very low rarefaction depth observed in leaf samples likely reduced sensitivity for detecting low‐abundance taxa and may have contributed to instability in diversity estimates. Leaf tissues are typically characterized by lower fungal biomass and a higher proportion of host‐derived DNA, conditions under which shorter amplicons such as ITS2 may provide more reliable amplification and improved specificity (Bellemain et al. 2010; Blaalid et al. 2013). In contrast, root‐associated communities tend to be more complex and variable, and may therefore be better captured by longer amplicons such as full‐length ITS, which can integrate a broader phylogenetic signal (Tedersoo et al. 2018; Runnel et al. 2022). Thus, for comparisons of community metrics across tissues, our study suggests that combining ITS2 and ITSf provides a more comprehensive understanding, whereas within‐tissue comparisons can be performed using a single marker, preferably ITS2 for leaves and ITSf for roots. The choice of bioinformatic classifier also influences downstream interpretation because assignment algorithms differ in sensitivity and conservativeness, particularly for short or poorly represented ITS sequences (Ye et al. 2006; Nilsson, Anslan, et al. 2019; Pauvert et al. 2019). In this study, taxonomic assignments were performed using a scikit‐learn–based classifier against the UNITE database, and differences in assignment depth among markers may partly reflect database representation and algorithm‐specific behavior. These considerations highlight the importance of reporting taxonomic confidence and improving reference database completeness for plant‐associated fungal communities. The use of a universal Eukaryotic reference database such as UNITE also enables the detection of non‐fungal taxa, including Viridiplantae (genus *Solanum*) and Stramenopila (genus *Phytophthora*). Due to the high degree of conservation in eukaryotic ITS regions, host‐derived sequences can dominate sequencing datasets, thereby reducing the effective resolution of microbial communities (Giubilei et al. 2026; Giangacomo et al. 2021; Taerum et al. 2020). To mitigate host contamination, several strategies can be employed, including the use of more selective primers or blocking approaches such as PNA clamps that inhibit amplification of host DNA (Borodušķe et al. 2023; Viotti et al. 2024; Giubilei et al. 2026). These approaches may be particularly relevant for improving fungal community characterization in plant‐associated systems. Patterns of community composition further highlight how marker choice influences the ecological characterization of plant‐associated microbiomes. Taxonomic profiles revealed that fungal communities were often dominated by a limited number of highly abundant taxa, with distinct patterns across markers and tissues. For instance, root‐associated communities showed strong dominance by specific genera such as *Plectosphaerella*, while other taxa including *Ilyonectria* and *Verticillium* contributed substantially to community structure. In leaf samples, community composition was often strongly influenced by the dominance of oomycetes such as *Phytophthora*, particularly in ITS1 and ITSf datasets, resulting in reduced community evenness. At the same time, additional genera such as *Coprinopsis*, *Neoascochyta*, and *Itersonilia* exhibited marker‐dependent patterns of detection and relative abundance. These compositional differences are consistent with the diversity patterns observed and reflect shifts not only in taxonomic richness but also in community evenness and dominance structure. Overlap analyses further demonstrated that only a limited fraction of ASVs was consistently shared among ITS markers, while a substantial proportion of taxa was uniquely detected by individual markers. This pattern indicates that each marker captures a partially distinct subset of the community, rather than providing a fully overlapping representation of the underlying microbiome. The relatively low overlap among markers helps explain the differences observed in community composition and diversity, particularly in root‐associated samples, where community complexity is higher. These findings further demonstrate that metabarcoding results are inherently marker‐dependent and should be interpreted as complementary rather than interchangeable views of microbial diversity. From a functional perspective, most detected taxa were assigned to ecological guilds characterized by flexible or context‐dependent roles, particularly endophytes with pathogenic potential. Genera such as *Ilyonectria* and *Verticillium* are commonly associated with multiple ecological lifestyles, including pathogenic, endophytic, and saprotrophic interactions, highlighting the ecological complexity of plant‐associated fungi (Klosterman et al. 2009; Molnár et al. 2025). This functional plasticity, together with the observed dominance patterns, suggests that shifts in relative abundance may have important ecological implications that are not fully captured by taxonomic diversity metrics alone. At the same time, the prevalence of multifunctional guilds highlights the limitations of assigning discrete ecological roles in metabarcoding studies, as functional classifications may oversimplify the context‐dependent nature of plant–fungal interactions (Tedersoo et al. 2022). Oomycetes belonging to the Stramenopila, mainly represented by *Phytophthora*, likely including *P. infestans* , the primary pathogen of potato, were consistently detected across tissues, despite the absence of obvious disease symptoms during sampling. Their detection was strongly marker‐dependent, as ITS1 and ITSf captured these taxa in both leaves and roots, whereas ITS2 failed to detect them. This pattern highlights that marker choice can influence not only taxonomic assignment depth but also the detection of ecologically and agronomically relevant taxa. Notably, the presence of taxa with known pathogenic potential further emphasizes that metabarcoding‐based assessments of plant‐associated microbiomes are inherently shaped by methodological choices, and that both taxonomic and functional inferences should be interpreted within this context. Although this study focused on a single crop species, the observed marker‐dependent patterns in taxonomic composition, diversity, and ecological representation are likely to extend across a wide range of plant–microbe systems. Similar trade‐offs in amplification efficiency, taxonomic coverage, and functional inference may therefore affect metabarcoding studies in other hosts and environments, underscoring the broader importance of marker selection for robust and ecologically meaningful community profiling. Several limitations should be considered when interpreting these results, including the use of pooled biological material, substantial host‐derived amplification, and the low sequencing depth observed in some leaf datasets. Consequently, the reported diversity patterns should be interpreted primarily as comparative assessments of marker‐associated compositional differences rather than definitive estimates of natural community structure. Taken together, these results highlight that no single ITS marker provides a complete representation of plant‐associated fungal communities, and that marker choice should be guided by both the target tissue and the specific ecological or applied research question. In this context, combining complementary markers such as ITS2 and full‐length ITS may offer a more comprehensive and balanced view of plant mycobiomes, improving the reliability of ecological interpretation and pathogen detection in plant‐associated systems.

## 5 Conclusion

This study demonstrates that ITS marker selection is a key factor when characterizing fungal communities in potato ( *S. tuberosum* ) tissues. ITS2 generally recovered more diverse and even community profiles in leaves, whereas ITSf recovered comparatively more even and compositionally broader root‐associated communities. In contrast, ITS1 showed high amplification efficiency but generated strongly skewed community profiles, highlighting a trade‐off between read recovery and compositional representativeness in this pathosystem. These findings emphasize that combining complementary markers such as ITS2 and ITSf provides a more comprehensive view of the potato mycobiome, whereas single‐marker approaches capture only a partial representation of the community structure. Thus, combining ITS2 and ITSf is recommended for cross‐tissue comparisons of community metrics, whereas within‐tissue comparisons can rely on a single marker, preferably ITS2 for leaves and ITSf for roots. In addition, continued improvements in reference database completeness and primer design will further enhance characterization of plant‐associated fungal communities for applications in potato crop management, pathogen surveillance, and microbiome‐based interventions. Future studies integrating complementary approaches will further improve the ecological and functional interpretation of plant‐associated microbial communities.

## Funding

The Gunnar and Ruth Björkman's Fund for Botanical Research in Norrland (Grant 516029133), awarded L.M. to perform the study during her Kempe Foundation Postdoctoral fellowship (Grant JCSMK23‐0066) awarded to B.R.A. The Knut and Alice Wallenberg Foundation (Grants KAW 2016.0352 and KAW 2020.0240) provided financial support for the UPSC infrastructure.

## Conflicts of Interest

The authors declare no conflicts of interest.

## Supporting information


**Figure S1:** Total reads obtained at the family, genus, and species taxonomic levels in potato leaves (top) and roots (bottom).

**Figure S2:** Relative abundance of ASVs at the phylum level in potato leaves (top) and roots (bottom), as detected with ITS1, ITS2, and full‐length ITS (ITSf) markers.

**Table S1:** Total number of reads, their taxonomic rank and ASVs count.

**Table S2:** Taxonomic classification of ASVs across ITS markers and plant tissues. Columns indicate ASV identity, sample information (tissue and marker), relative abundance (%), replicate grouping, and taxonomic assignment.

**Table S3:** Relative abundance of the 20 most abundant genera among ITS regions in leaf and root communities.

**Table S4:** ASVs at the genus level across leaf and root samples according to the ITS region considered.

## Data Availability

The data that support the findings of this study are openly available in NCBI SRA Database at https://www.ncbi.nlm.nih.gov/sra/?term=PRJNA1330153, reference number PRJNA1330153.

## References

[pei370168-bib-0001] Abarenkov, K. , R. H. Nilsson , K. H. Larsson , et al. 2024. “The UNITE Database for Molecular Identification and Taxonomic Communication of Fungi and Other Eukaryotes: Sequences, Taxa and Classifications Reconsidered.” Nucleic Acids Research 52, no. D1: D791–D797. 10.1093/nar/gkad1039. 37953409 PMC10767974

[pei370168-bib-0002] Albrectsen, B. R. , L. Björkén , A. Varad , et al. 2010. “Endophytic Fungi in European Aspen ( *Populus tremula* ) Leaves—Diversity, Detection, and a Suggested Correlation With Herbivory Resistance.” Fungal Diversity 41: 17–28. 10.1007/s13225-009-0011-y.

[pei370168-bib-0003] Bellemain, E. , T. Carlsen , C. Brochmann , E. Coissac , P. Taberlet , and H. Kauserud . 2010. “ITS as an Environmental DNA Barcode for Fungi: An In Silico Approach Reveals Potential PCR Biases.” BMC Microbiology 10: 1–9. 10.1186/1471-2180-10-189. 20618939 PMC2909996

[pei370168-bib-0004] Blaalid, R. , S. Kumar , R. H. Nilsson , K. Abarenkov , P. M. Kirk , and H. Kauserud . 2013. “ITS1 Versus ITS2 as DNA Metabarcodes for Fungi.” Molecular Ecology Resources 13: 218–224. 10.1111/1755-0998.12065. 23350562

[pei370168-bib-0005] Blackwell, M. 2011. “The Fungi: 1, 2, 3… 5.1 Million Species?” American Journal of Botany 98, no. 3: 426–438. 10.3732/ajb.1000298. 21613136

[pei370168-bib-0006] Bolyen, E. , J. R. Rideout , M. R. Dillon , et al. 2019. “Reproducible, Interactive, Scalable and Extensible Microbiome Data Science Using QIIME 2.” Nature Biotechnology 37: 852–857. 10.1038/s41587-019-0209-9. PMC7015180 31341288

[pei370168-bib-0007] Borodušķe, A. , J. Ķibilds , D. Fridmanis , et al. 2023. “Does Peptide‐Nucleic Acid (PNA) Clamping of Host Plant DNA Benefit ITS1 Amplicon‐Based Characterization of the Fungal Endophyte Community?” Fungal Ecology 61: 101181. 10.1016/j.funeco.2022.101181.

[pei370168-bib-0008] Cardacino, A. , T. Tastekin , F. Brugneti , M. Cirilli , A. Mazzaglia , and S. Turco . 2025. “Peach Buds’ Microbiome Profiling Reveals Cultivar‐Specific Signatures Associated With TCSB Susceptibility.” Stress 5: 60. 10.3390/stresses5030060.

[pei370168-bib-0010] Chrominski, P. , U. Carlson‐Nilsson , A. Palmé , H. G. Kirk , Å. Asdal , and L. Ansebo . 2024. “Genetic Markers Identify Duplicates in Nordic Potato Collections.” Frontiers in Plant Science 15: 1405314. 10.3389/fpls.2024.1405314. 39253569 PMC11381411

[pei370168-bib-0011] Coomber, M. G. , B. J. Nielsen , G. C. Nielsen , M. Pautasso , and E. M. Hansen . 2023. “Genetic Diversity and Population Structure of *Phytophthora infestans* in Denmark: Evidence for Predominant Clonal Reproduction.” PLoS One 18, no. 3: e0283540. 10.1371/journal.pone.0283540. 37011062 PMC10069789

[pei370168-bib-0012] Gardes, M. , and T. D. Bruns . 1993. “ITS Primers With Enhanced Specificity for Basidiomycetes Application to the Identification of Mycorrhizae and Rusts.” Molecular Ecology 2, no. 2: 113–118. 10.1111/j.1365-294X.1993.tb00005.x. 8180733

[pei370168-bib-0013] Gautam, A. K. , R. K. Verma , S. Avasthi , et al. 2022. “Current Insight Into Traditional and Modern Methods in Fungal Diversity Estimates.” Journal of Fungi 8, no. 3: 226. 10.3390/jof8030226. 35330228 PMC8955040

[pei370168-bib-0014] Giangacomo, C. , M. Mohseni , L. Kovar , and J. G. Wallace . 2021. “Comparing DNA Extraction and 16S rRNA Gene Amplification Methods for Plant‐Associated Bacterial Communities.” Phytobiomes Journal 5, no. 2: 190–201. 10.1094/PBIOMES-07-20-0055-R.

[pei370168-bib-0015] Giubilei, I. , S. Turco , L. Mahawar , B. R. Albrectsen , and A. Mazzaglia . 2026. “Clearing the Noise: Seasonal Dynamics of Endophytic Bacteria in *Fagus sylvatica* Leaves Revealed by PNA Clamps.” Physiologia Plantarum 178, no. 3: e70897. 42036304 10.1111/ppl.70897 PMC13110927

[pei370168-bib-0016] Hawksworth, D. L. , and R. Lücking . 2017. “Fungal Diversity Revisited: 2.2 to 3.8 Million Species.” Microbiology Spectrum 5. 10.1128/microbiolspec.funk-0052-2016. PMC11687528 28752818

[pei370168-bib-0017] Hyde, K. D. , J. Xu , S. Rapior , et al. 2019. “The Amazing Potential of Fungi: 50 Ways We Can Exploit Fungi Industrially.” Fungal Diversity 97: 1–136. 10.1007/s13225-019-00430-9.

[pei370168-bib-0018] Klosterman, S. J. , Z. K. Atallah , G. E. Vallad , and K. V. Subbarao . 2009. “Diversity, Pathogenicity, and Management of *Verticillium* Species.” Annual Review of Phytopathology 47: 39–62. 10.1146/annurev-phyto-080508-081748. 19385730

[pei370168-bib-0019] Lindahl, B. D. , R. H. Nilsson , L. Tedersoo , et al. 2013. “Fungal Community Analysis by High‐Throughput Sequencing of Amplified Markers ‐ a User's Guide.” New Phytologist 199, no. 1: 288–299. 10.1111/nph.12243. 23534863 PMC3712477

[pei370168-bib-0020] Mahawar, L. , A. Mishra , A. Tsitouri , and B. R. Albrectsen . 2026. “Straw Mulching Differentially Shapes the Structure and Function of Below‐Ground Bacterial Communities in Potato Depending on eDNA Source and Cultivar.” Plant‐Environment Interactions 7: e70131. 10.1002/pei3.70131. 41727921 PMC12921270

[pei370168-bib-0021] Mbareche, H. , M. Veillette , and G. J. Bilodeau . 2021. “In Silico Study Suggesting the Bias of Primers Choice in the Molecular Identification of Fungal Aerosols.” Journal of Fungi 7, no. 2: 99. 10.3390/jof7020099. 33573216 PMC7911573

[pei370168-bib-0022] Mittelstrass, J. , R. Heinzelmann , R. Eschen , et al. 2025. “Metabarcoding With Illumina and Oxford Nanopore Technologies Provides Complementary Insights Into Tree Seed Mycobiota.” Environmental Microbiomes 20, no. 1: 53. 10.21203/rs.3.rs-5368169/v1. PMC12090628 40390141

[pei370168-bib-0023] Molnár, N. , D. Szabó , A. Geiger , J. Geml , K. Z. Váczy , and Z. Karácsony . 2025. “Isolation and Characterization of the Phytopathogenic Fungus *Ilyonectria liriodendri* From Persimmon as a New Susceptible Host.” PLoS One 20: e0339616. 10.1371/journal.pone.0339616. 41452856 PMC12742776

[pei370168-bib-0024] Nilsson, R. H. , S. Anslan , M. Bahram , C. Wurzbacher , P. Baldrian , and L. Tedersoo . 2019. “Mycobiome Diversity: High‐Throughput Sequencing and Identification of Fungi.” Nature Reviews Microbiology 17, no. 2: 95–109. 10.1038/s41579-018-0116-y. 30442909

[pei370168-bib-0025] Nilsson, R. H. , K. H. Larsson , A. F. S. Taylor , et al. 2019. “The UNITE Database for Molecular Identification of Fungi: Handling Dark Taxa and Parallel Taxonomic Classifications.” Nucleic Acids Research 47, no. D1: D259–D264. 10.1093/nar/gky1022. 30371820 PMC6324048

[pei370168-bib-0026] Op De Beeck, M. , B. Lievens , P. Busschaert , G. De Cutijpere , J. Vangronsveld , and J. V. Colpaert . 2014. “Comparison and Validation of ITS Primer Pairs Useful for Fungal Metabarcoding Studies.” PLoS One 9, no. 6: e97629. 10.1371/journal.pone.0097629. 24933453 PMC4059633

[pei370168-bib-0027] Pauvert, C. , M. Buée , V. Laval , et al. 2019. “Bioinformatics Matters: The Accuracy of Plant and Soil Fungal Community Data Is Highly Dependent on the Metabarcoding Pipeline.” Fungal Ecology 41: 23–33. 10.1016/j.funeco.2019.03.005.

[pei370168-bib-0028] Põlme, S. , K. Abarenkov , R. Henrik Nilsson , et al. 2020. “FungalTraits: A User‐Friendly Traits Database of Fungi and Fungus‐Like Stramenopiles.” Fungal Diversity 105: 1–16. 10.1007/s13225-020-00466-2.

[pei370168-bib-0029] Runnel, K. , K. Abarenkov , O. Copoț , et al. 2022. “DNA Barcoding of Fungal Specimens Using PacBio Long‐Read High‐Throughput Sequencing.” Molecular Ecology Resources 22: 2871–2879. 10.1111/1755-0998.13663. 35666173

[pei370168-bib-0030] Runnel, K. , L. Tedersoo , F. S. Krah , et al. 2025. “Toward Harnessing Biodiversity–Ecosystem Function Relationships in Fungi.” Trends in Ecology & Evolution 40, no. 2: 180–190. 10.1016/j.tree.2024.10.004. 39532622

[pei370168-bib-0031] Schoch, C. L. , K. A. Seifert , S. Huhndorf , et al. 2012. “Nuclear Ribosomal Internal Transcribed Spacer (ITS) Region as a Universal DNA Barcode Marker for.” Proceedings of the National Academy of Sciences of the United States of America 109, no. 16: 6241–6246. 10.1073/pnas.1117018109. 22454494 PMC3341068

[pei370168-bib-0032] Seena, S. , C. Pascoal , L. Marvanová , and F. Cássio . 2010. “DNA Barcoding of Fungi: A Case Study Using ITS Sequences for Identifying Aquatic Hyphomycete Species.” Fungal Diversity 44: 77–87. 10.1007/s13225-010-0056-y.

[pei370168-bib-0033] Siddique, A. B. , B. R. Albrectsen , H. Ilbi , and A. B. Siddique . 2022. “Optimization of Protocol for Construction of Fungal ITS Amplicon Library for High‐Throughput Illumina Sequencing to Study the Mycobiome of Aspen Leaves.” Applied Sciences 12, no. 3: 1136. 10.3390/app12031136.

[pei370168-bib-0034] Sun, X. , and L.‐D. Guo . 2012. “Endophytic Fungal Diversity: Review of Traditional and Molecular Techniques.” Mycology 3, no. 1: 65–76. 10.1080/21501203.2012.656724.

[pei370168-bib-0035] Taerum, S. J. , B. Steven , D. J. Gage , and L. R. Triplett . 2020. “Validation of a PNA Clamping Method for Reducing Host DNA Amplification and Increasing Eukaryotic Diversity in Rhizosphere Microbiome Studies.” Phytobiomes Journal 4, no. 4: 291–302. 10.1094/PBIOMES-05-20-0040-TA.

[pei370168-bib-0036] Taylor, D. , W. Walters , N. Lennon , et al. 2016. “Accurate Estimation of Fungal Diversity and Abundance Through Improved Lineage‐Specific Primers Optimized for Illumina Amplicon Sequencing.” Applied and Environmental Microbiology 82: 7217–7226. 10.1128/AEM.02576-16. 27736792 PMC5118932

[pei370168-bib-0038] Tedersoo, L. , S. Anslan , M. Bahram , et al. 2015. “Shotgun Metagenomes and Multiple Primer Pair‐Barcode Combinations of Amplicons Reveal Biases in Metabarcoding Analyses of Fungi.” MycoKeys 10: 1–43. 10.3897/mycokeys.10.4852.

[pei370168-bib-0037] Tedersoo, L. , S. Anslan , M. Bahram , U. Kõljalg , and K. Abarenkov . 2020. “Identifying the ‘Unidentified’ Fungi: A Global‐Scale Long‐Read Third‐Generation Sequencing Approach.” Fungal Diversity 103: 273–293. 10.1007/s13225-020-00456-4.

[pei370168-bib-0039] Tedersoo, L. , M. Bahram , L. Zinger , et al. 2022. “Best Practices in Metabarcoding of Fungi: From Experimental Design to Results.” Molecular Ecology 31, no. 10: 2769–2795. 10.1111/mec.16460. 35395127

[pei370168-bib-0040] Tedersoo, L. , A. Tooming‐Klunderud , and S. Anslan . 2018. “PacBio Metabarcoding of Fungi and Other Eukaryotes: Errors, Biases and Perspectives.” New Phytologist 217, no. 3: 1370–1385. 10.1111/nph.14776. 28906012

[pei370168-bib-0041] Toju, H. , A. S. Tanabe , S. Yamamoto , H. Sato , and O. Lespinet . 2012. “Highcoverage ITS Primers for the DNA‐Based Identification of Ascomycetes and Basidiomycetes in Environmental Samples.” PLoS One 7, no. 7: e40863. 10.1371/journal.pone.0040863. 22808280 PMC3395698

[pei370168-bib-0042] Turco, S. , F. Brugneti , I. Giubilei , et al. 2024. “A Bud's Life: Metabarcoding Analysis to Characterise Hazelnut Big Buds Microbiome Biodiversity.” Microbiological Research 287: 127851. 10.1016/j.micres.2024.127851. 39094393

[pei370168-bib-0043] Veteläinen, M. , E. Gammelgård , and J. P. T. Valkonen . 2005. “Diversity of Nordic Landrace Potatoes ( *Solanum tuberosum* L.) Revealed by AFLPs and Morphological Characters.” Genetic Resources and Crop Evolution 52: 999–1010. 10.1007/s10722-003-6129-y.

[pei370168-bib-0044] Viotti, C. , M. Chalot , P. G. Kennedy , et al. 2024. “Primer Pairs, PCR Conditions, and Peptide Nucleic Acid Clamps Affect Fungal Diversity Assessment From Plant Root Tissues.” Mycology 15, no. 2: 255–271. 10.1080/21501203.2023.2301003. 38813472 PMC11132971

[pei370168-bib-0046] White, T. J. , T. Bruns , S. J. W. T. Lee , and J. Taylor . 1990. “Amplification and Direct Sequencing of Fungal Ribosomal RNA Genes for Phylogenetics.” PCR Protocols: A Guide to Methods and Applications 18, no. 1: 315–322. 10.1016/B978-0-12-372180-8.50042-1.

[pei370168-bib-0047] Wijayawardene, N. , M. Bahram , I. Sánchez‐Castro , et al. 2021. “Current Insight Into Culture‐Dependent and Culture‐Independent Methods in Discovering Ascomycetous Taxa.” Journal of Fungi 7: 703. 10.3390/jof7090703. 34575741 PMC8467358

[pei370168-bib-0048] Winand, R. , E. D'hooge , A. Van Uffelen , et al. 2025. “Investigating Fungal Diversity Through Metabarcoding for Environmental Samples: Assessment of ITS1 and ITS2 Illumina Sequencing Using Multiple Defined Mock Communities With Different Classification Methods and Reference Databases.” BMC Genomics 26: 729. 10.1186/s12864-025-11917-y. 40770684 PMC12329927

[pei370168-bib-0049] Ye, J. , S. McGinnis , and T. L. Madden . 2006. “BLAST: improvements for better sequence analysis.” Nucleic acids research 34, no. suppl_2: W6–W9. 10.1093/nar/gkl164. 16845079 PMC1538791

