# Supplementary figures and images for: Comparative Metabarcoding of ITS1, ITS2, and Full‐Length ITS Reveals Marker‐ and Tissue‐Specific Variation in Fungal Community Profiling in Potato
Source: Plant Environ Interact. 2026 Jun 2;7(3):e70168. doi: 10.1002/pei3.70168 (PMC13239480; doi:10.1002/pei3.70168)

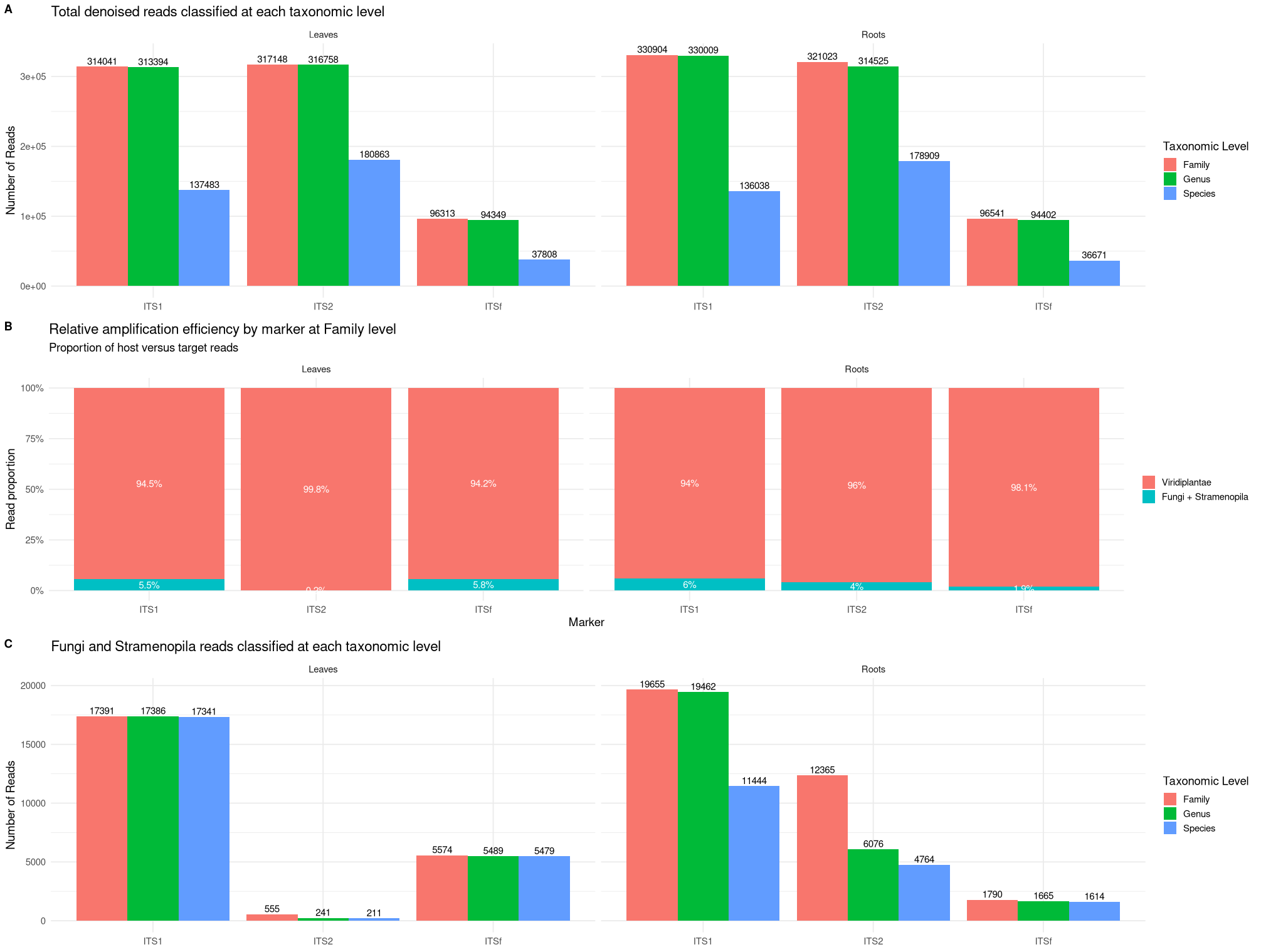

Supplement: Supplementary file 1 — Figure S1: Total reads obtained at the family, genus, and species taxonomic levels in potato leaves (top) and roots (bottom). [file PEI3-7-e70168-s002.tiff]

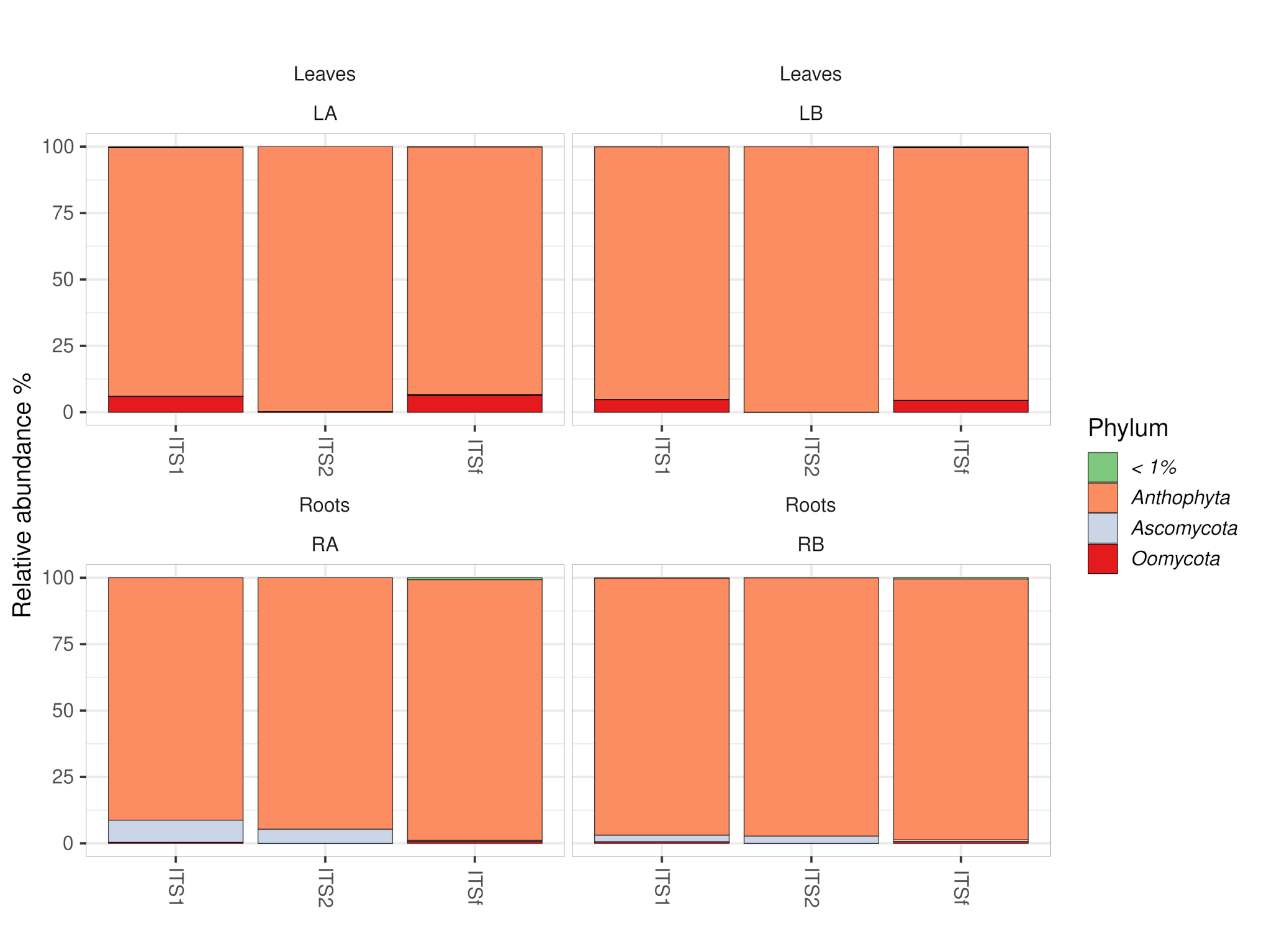

Supplement: Supplementary file 2 — Figure S2: Relative abundance of ASVs at the phylum level in potato leaves (top) and roots (bottom), as detected with ITS1, ITS2, and full‐length ITS (ITSf) markers. [file PEI3-7-e70168-s001.tif]
